# Influence of PDA Coating on the Structural, Optical and Surface Properties of ZnO Nanostructures

**DOI:** 10.3390/nano10122438

**Published:** 2020-12-06

**Authors:** Daina Damberga, Viktoriia Fedorenko, Kārlis Grundšteins, Şahin Altundal, Andris Šutka, Arunas Ramanavičius, Emerson Coy, Radosław Mrówczyński, Igor Iatsunskyi, Roman Viter

**Affiliations:** 1 Institute of Atomic Physics and Spectroscopy, University of Latvia, Jelgavas 3, LV-1004 Riga, Latvia; daina.damberga@fizmati.lv (D.D.); viktoriia.fedorenko@lu.lv (V.F.); Karlis.Grundsteins@rtu.lv (K.G.); sahinaltundal@gmail.com (Ş.A.); Andris.Sutka@rtu.lv (A.Š.); arunas.ramanavicius@chf.vu.lt (A.R.); 2 Research Laboratory of Functional Materials Technologies, Faculty of Materials Science and Applied Chemistry, Riga Technical University, Paula Valdena 3/7, LV-1048 Riga, Latvia; 3 Laboratory of Nanotechnology, State Research Institute Center for Physical Sciences and Technology, Sauletekio ave.3, LT-10257 Vilnius, Lithuania; 4 NanoBioMedical Centre, Adam Mickiewicz University in Poznan, Wszechnicy Piastowskiej 3, 61-614 Poznan, Poland; coyeme@amu.edu.pl (E.C.); rm53520@amu.edu.pl (R.M.); 5 Center for Collective Use of Scientific Equipment, Sumy State University, 31, Sanatornaya st., 40018 Sumy, Ukraine

**Keywords:** ZnO–polydopamine nanocomposites, fundamental properties, optical sensors

## Abstract

Polydopamine (PDA) is a new biocompatible material, which has prospects in biomedical and sensor applications. Due to functional groups, it can host wide range of biomolecules. ZnO nanostructures are well known templates for optical sensors and biosensors. The combination of ZnO and PDA results in a change of optical properties of ZnO–PDA composites as a shift of photoluminescence (PL) peaks and PL quenching. However, to date, the effect of the PDA layer on fundamental properties of ZnO–PDA nanostructures has not been studied. The presented paper reports on optical and surface properties of novel ZnO–PDA nanocomposites. PDA layers were chemically synthesized on ZnO nanostructures from different solution concentrations of 0.3, 0.4, 0.5 and 0.7 mg/mL. Structure, electronic and optical properties were studied by SEM, Raman, FTIR, diffuse reflectance and photoluminescence methods. The Z-potential of the samples was evaluated in neutral pH (pH = 7.2). The response of the samples towards poly-l-lysine adsorption, as a model molecule, was studied by PL spectroscopy to evaluate the correlation between optical and surface properties. The role of the PDA concentration on fundamental properties was discussed.

## 1. Introduction

Inorganic–organic core shell composite materials attract the attention of researchers due to their optical, electrical and sensitive properties [1]. The organic shell on the inorganic core increases the surface properties of the composite, by introducing active functional groups, additional absorption and/or emission peaks. Tailored optical properties of the composites are important for possible applications in sensors, optical coatings and photocatalysis [2]. 

Among the different inorganic and organic nanomaterials, a zinc oxide (ZnO) and polydopamine (PDA) composition might have advanced optical properties. ZnO is a well known material with a wide band gap and strong room temperature photoluminescence [3]. It is often used as a template in sensor, coating and photocatalytic applications [4]. 

PDA has prospects in biomedical applications due to its biocompatibility and proteins affinity. Polydopamine is formed within the self-polymerization of dopamine in basic solution [5,6]. Thin layers and nanoparticles of PDA could be formed with a controlled shape and properties [7,8,9], which have been shown to be quite promising for photocatalysis, especially when combined with photoactive nanomaterials [10]. In general, it is clear that the deposition time and concentration of dopamine define the fundamental properties of PDA nanostructures [5,9].

Previously, it was shown that a PDA layer could be deposited on a ZnO surface by chemical deposition to form ZnO/PDA nanocomposites [8,11,12,13]. PDA deposition resulted in a shift of the band gap of ZnO and a change of photoluminescence peak position in ZnO/PDA nanostructures [6]. The investigation of the temperature and power dependences of ZnO and ZnO/PDA nanocomposites were studied in the range of 77–300 K. Defect concentrations, quantum efficiency and activation energies in ZnO and ZnO/PDA nanostructures were studied. It was shown that PDA deposition resulted in a decrease in the defect concentrations in ZnO, particularly, oxygen vacancies from 1.5 × 10^15^ cm^−3^ to 1.2 × 10^14^ cm^−3^. As a result, the quantum efficiency of photoluminescence decreased from 0.28 to 0.12 for ZnO/PDA. PDA deposition resulted in the decreased activation energies in ZnO/PDA. Exciton binding energy decreased from 0.053 to 0.044 eV in ZnO/PDA. The mechanism of the ZnO/PDA interface formation was proposed. The main assumption was based on the fact that the PDA formed a binding of hydroxyl groups with ZnO surface oxygen vacancies. As a result, the depletion layer width in ZnO increased. This induced changes in the optical properties of the composites (quenching of photoluminescence (PL), decrease in activation energies, etc.). 

In spite of the reported achievements, the role of the PDA layer in the optical properties of ZnO in ZnO/PDA nanocomposites is not fully understood yet. Particularly, the effect of PDA concentration on optical properties of the composites at fixed deposition time has not been studied yet. Therefore, in the present paper, we report on the investigation of the structural, electronic and optical properties of ZnO/PDA, where the PDA layer was formed at different concentrations of initial precursors. The role of PDA layer fabrication conditions in tailoring the optical properties and surface properties of ZnO/PDA will be discussed.

## 2. Materials and Methods 

### 2.1. Materials

Zinc acetate dehydrate, hexamethylenetetramine, 2-propanol (IPA), ethanolamine, sodium sulphate, zinc nitrate hexahydrate 0.1% (*w/v*) and poly-L-lysine solution in H_2_O were obtained from Sigma Aldrich (Riga, Latvia), dopamine hydrochloride 99%, and tris(hydroxmethyl)amino- methane 99% were purchased from Alfa Aesar (Poland), and were used without additional purification. The glass substrates (10 mm × 10 mm) were cleaned by successive sonication with deionized water and isopropyl alcohol for 10 min, with proper drying prior to the final use. The oxygen plasma treatment for 15 min was performed in order to eliminate organic traces.

### 2.2. Fabrication of ZnO Nanorods

ZnO nanorods (ZnONRs) were deposited by hydro- thermochemical method as described in Viter et al. [14,15]. The initial ZnO seed layer was prepared on glass by the drop casting of 20 mL of 1 mg mL^−1^ zinc acetate methanol solution, followed by annealing at 350 °C for 1 h. The glass substrates with ZnO seed layers were incubated for 4 h in 50 mM of zinc nitrate and 50 mM of hexamethylenetetramine containing solution in water at 95 °C. A hydrothermal growth of ZnONRs was performed. The samples were washed by deionized water and dried at room temperature.

### 2.3. Forming of a PDA Layer Over ZnO-NR

As deposited glass substrates (size 10 × 10 mm^2^) with ZnONRs were immersed into a Tris buffer (10 mM, pH 8.5, 50 mL) with various dopamine concentrations in the range of 0.3–0.7 mg mL^−1^ at room temperature for a deposition time of 2 h, unless stated otherwise. In the next step, the samples were removed and rinsed with Milli-Q water and dried with nitrogen (N_2_) stream.

### 2.4. Characterization

The structural properties of the ZnONRs–PDA nanostructures were investigated by XRD (PANAlytical Xpert-PRO diffractometer equipped with a Pixel 3D detector using Ni-filtered Cu Kα radiation 45 kV/40 mA), SEM (Zeiss Evo HD15 SEM from Zeiss Ltd. (Jena, Germany)), HR-TEM (JEOL ARM 200F) high-resolution transmission electron microscope (200 kV) with an EDX and EELS detector, Raman scattering measurements were performed using a Renishaw micro-Raman spectrometer equipped with a confocal microscope (Leica) and FTIR spectroscopy, using a FTIR-ATR spectrophotometer ‘Frontier’ from Perkin Elmer (Waltham, MA, USA). Optical properties were studied by diffuse reflectance spectroscopy, using a UV–Vis light source, integrating sphere, HR2000+ fiber spectrometer from Ocean Optics (Dunedin, FL, USA) and room temperature photoluminescence spectroscopy (325 nm LED, output power 5 mw, Roithner, Austria). For the z-potential measurements, pristine ZnO and ZnO–PDA samples were dispersed in deionized water and were then measured at pH = 6.5 with by the Zetasizer Nano ZS (Malvern, Panalytical).

## 3. Results

**Figure 1:**
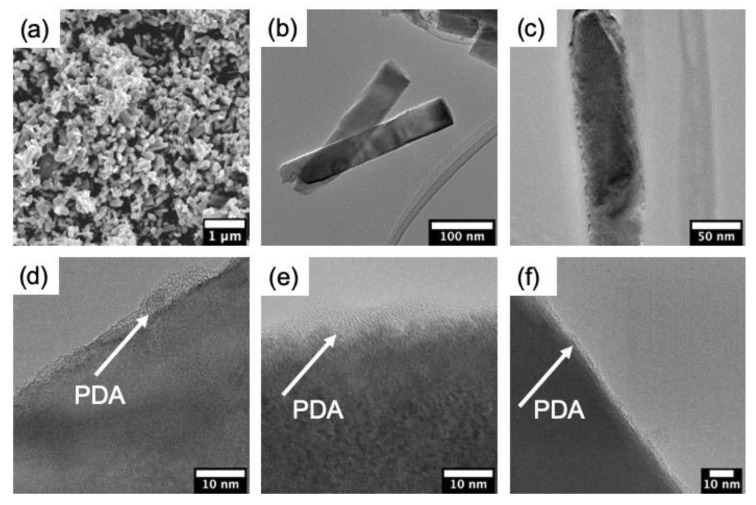
(**a**) The SEM and (**b**) the TEM images of pristine ZnONR; (**c**) the TEM images of a separate ZnO/polydopamine (PDA) nanorod (0.5 mg/mL) and HRTEM images of ZnO/PDA nanorods (NRs) at (**d**) 0.3, (**e**) 0.5 and (**f**) 0.7 mg/mL.

The morphology of pristine ZnONR and produced ZnO–PDA samples was investigated by TEM and SEM. Figure 1a demonstrates the typical SEM image of ZnONR on the glass substrate. As is clearly seen, ZnONRs are uniformly distributed over the surface. The average length of ZnONRs is in the range of 200–600 nm and approximately 60 ± 10 nm in diameter (Figure 1b). TEM images of ZnO–PDA showed a clear indication of PDA coating (an average thickness of about 3–5 nm) over ZnONRs (Figure 1c–f), thereby confirming the formation of ZnO–PDA core-shell nanostructures. However, it is seen that the conformality of the PDA layer depends on the dopamine concentration. One may observe the non-uniform PDA layer for a low-concentrated dopamine solution (Figure 1d). Meanwhile, the conformal and uniform PDA layer is observed for the samples produced at high concentrations of dopamine (Figure 1f). It is well known that the PDA layer thickness depends on the time [6]. However, the concentration might affect the polymerization speed and other dynamics of the layer deposition. This might suggest that the “high” concentration was the ideal for the polymerization of the PDA layer on ZnONR.

**Figure 2:**
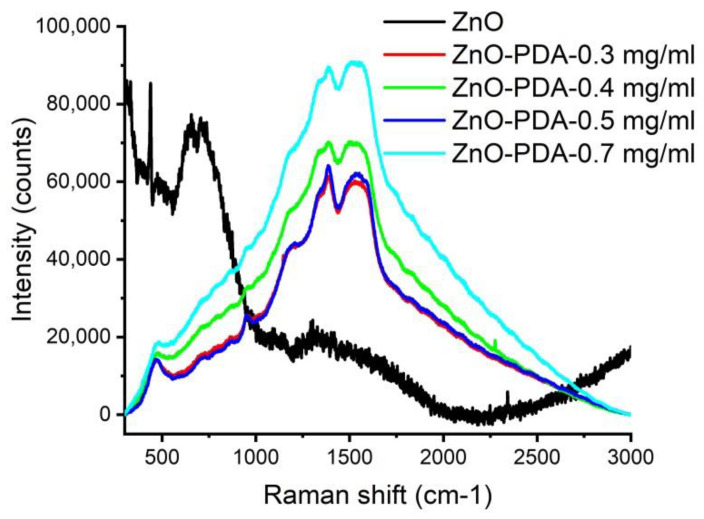
Raman spectra of ZnO with different PDA concentrations. ZnO spectra multiplied by a factor of 40.

In order to determine the composition of the produced nanocomposites, Raman spectroscopy, as a very sensitive method, was also used [16]. The Raman spectra of ZnO and ZnO–PDA nanorods are shown in Figure 2. As prepared ZnONRs showed peaks at 333, 376, and 435 cm^−1^, related to E_2_(high)–E_2_(low) mode, A_1_(TO) polar optical phonon mode, and E_2_ (high) non-polar mode, respectively [17]. The observed peak at 435 cm^−1^ (E_2_ high mode) corresponds to the wurtzite phase of ZnO, as was also confirmed by XRD. This mode is associated with a vibration of oxygen atoms in the crystal lattice. A Raman peak at 586 cm^−1^ could be related to structural disorders (such as oxygen vacancies, Zn interstitial, etc.) [18]. ZnO–PDA nanostructures showed peaks at 467, 541, 734, 954, 1183, 1334, 1391, 1478, 1559 cm^−1^. Peaks at 468 cm^−1^ and 954 cm^−1^ correspond to Zn–OH and O−H out-of-plane deformation mode. The peak at 541 cm^−1^ corresponds to the defect surface mode of ZnO [19]. Raman peaks at 1183, 1334, 1391, 1478, 1559 cm^−1^ corresponding to PDA [20]. According to the PDA Raman peak positions, as reported earlier, we can consider that peaks, located at 1181 cm^−1^ are C–OH phenolic stretching or the stretching of the catechol C–O bond, 1341 and 1393 cm^−1^ are the aromatic C–N stretching mode of the indole structure, 1478 and 1559 cm^−1^, correspond to C=C and C–C vibrations, respectively. The increase in the PDA concentration does not show a significant effect on the vibration energies.

Wide peaks at 466 and 1596 cm^−1^ were deconvoluted by two components via Lorentzian fitting and showing the following values: 457, 482, 1525 and 1596 cm^−1^. The analysis showed the good correspondence of the present peaks—1206, 1528 and 1595, and 1386 cm^−1^ to the PDA Raman modes, which related to C–OH or/and C–O, C=C, C–N, N–H and C=O vibrations, respectively [20].

The peak at 457 cm^−1^ relates to ZnO, whereas peaks at 482 and 954 cm^−1^ correspond to Zn–OH and O−H out-of-plane deformation mode. The increase in the PDA concentration does not show a significant effect on the vibration energies.

**Figure 3:**
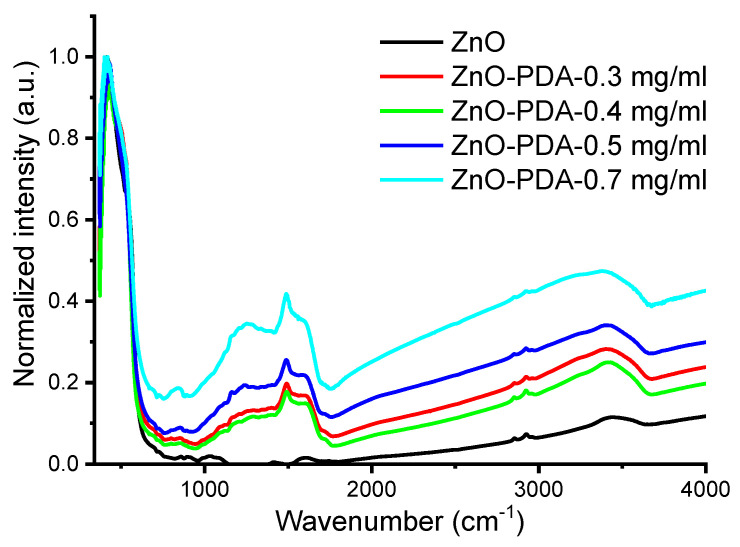
FTIR spectra of ZnO with 1 h PDA deposition.

FTIR spectra of the ZnO and ZnO–PDA nanorods are shown in Figure 3. As deposited ZnO nanorods had a specific peak at 400 cm^−1^ and a shoulder at 560 cm^−1^, which correspond to ZnO vibrational modes [6]. The forming of PDA stimulated the occurrence of new peaks in the range of 1100–2000 cm^−1^. The increase in the concentration of PDA resulted in an increase in the absorption peak value due to the increase in PDA thickness. PDA peaks are located at 1288, 1492, 1607, 3362 cm^−1^, which correspond to C–O, C=N or/and C=C, C=O and –OH or/and N–H vibrational modes [21]. The forming of PDA-based composites results in a shift of FTIR peaks. Compared to the pure ZnO FTIR spectra, the formation of a PDA layer around ZnO led to a shift in the FTIR peak positions of 12–20 cm^−1^ to the lower values of wavenumbers.

**Figure 4:**
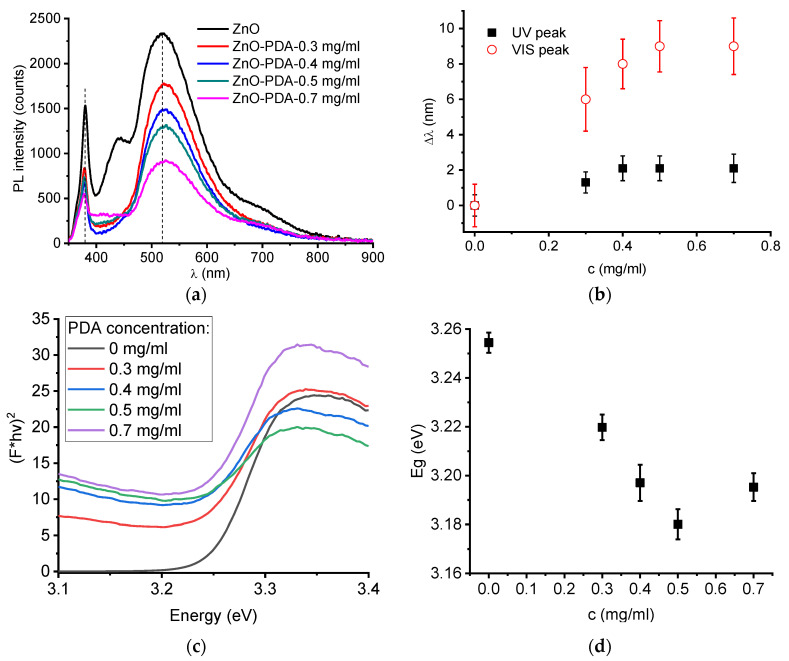
Optical characterization of ZnO–PDA nanorods with various PDA concentrations: (**a**) PL spectra; (**b**) change of the photoluminescence (PL) peak position in the UV and visible range; (**c**) graph for the band gap estimation; (**d**) band gap vs. dopamine concentration.

Photoluminescence (PL) is a simple method for surface defect characterization [3,22,23]. It shows defects, responsible for certain PL bands. The analysis of PL emission vs. excitation power allows to evaluate the defect concentration and the quantum efficiency [3,22,23]. Optical characterization was performed by using PL and diffuses reflectance spectroscopy. The room temperature photoluminescence of ZnO–PDA nanorods is shown in Figure 4a. ZnO–PDA nanostructures had two peaks, located in UV and visible ranges. These peaks correspond to exciton and defect emissions [6,24]. Analysis PL spectra showed changes of the spectra, affected by the PDA layer. After the deposition of the PDA layer, UV peak position (378 nm) shifted towards a shorter wavelength, whereas the visible peak position shifted longer wavelengths (Figure 4a). Absolute values of difference of peak positions between ZnO and ZnO–PDA nanostructures in the UV and visible range are plotted in Figure 4b. From Figure 4b, it was found that the peak shift value increased with PDA concentration. The saturation of the shift values was observed for both peaks at a dopamine concentration of 0.7 mg/mL. The forming of the PDA layer on the ZnO surfaces resulted in PL quenching. The PL quenching effect was proportional to PDA concentration. 

The band gap values of ZnO–PDA were graphically calculated in the linear part of the absorption edge (Figure 4c). The band gap dependence of ZnO–PDA nanostructures vs. dopamine concentration (Figure 4d) showed saturation at concentrations of 0.5–0.7 mg/mL. The obtained values are lower than the typical value for ZnO single crystal (E_g_ = 3.37 eV). The shift of the PL peaks and reduction in the band gap point to the change of surface properties of ZnO after PDA deposition.

The quenching of photoluminescence after PDA deposition can be partially explained by the formation of a thicker PDA layer over ZnO, confirmed by TEM observations. The increase in the PDA concentration results in an enhanced light absorption by the PDA layer. However, the enhanced light absorption does not explain the change of UV and visible peaks position. 

Previously, we reported on the possible forming of the ZnO–PDA interface and the effects for optical properties of ZnO/PDA nanostructures for one fixed PDA concentration [6]. The proposed mechanisms of the change of the optical properties were the following:

- ZnO oxygen vacancies were involved in dopamine polymerization as absorbance centers;
- Local field was formed between ZnO and PDA;
- Extension of depletion layer in ZnO appeared.

All these reduced the concentration of the PL emission centers and emission quantum efficiency. 

In the present work, the photoluminescence properties of ZnO–PDA nanostructures, PL intensity and peak positions, are affected by PDA concentration. It is expected that the PL quenching will remain at higher PDA concentrations. The saturation of the values, equal to a peak shift (nm) at a 0.7 mg/mL PDA concentration, can be explained by the fixed concentrations of electrons, emission centers and ZnO dimensions. This means that the depletion layer in ZnO nanorods has a restricted value, which does not change with a higher increase in PDA concentration. 

The band gap of ZnO–PDA nanostructures decreased with the increase in the PDA concentration. As reported before, this difference could be due to the forming of point defects and charge transfer from ZnO towards the PDA layer [25]. Similar effects were observed in acceptor-doped ZnO nanostructures or Schottky-type junction where the electron transfer from ZnO to the doping agent/or altering layer took place [26]. The saturation of the band gap value against the PDA concentration is in good correlation with the observed photoluminescence properties. The band gap saturation is reached due to the restriction of the PDA layer effect to the charge transfer or extension of the depletion layer in ZnO.

In the final stage, the z-potential of ZnONR and ZnO–PDA samples, which is indicative of the surface charge, and demonstrates the applicability of produced composites towards biosensing application, were measured. The z-potential of ZnONRs was +28.8 ± 7.1 mV, showing the positive charge of the surface. While the z-potential of ZnO–PDA samples was about −27.5 ± 5.3 mV. This means that after the ZnO surface modification by PDA, the ZnO–PDA samples may be used as an effective biosensing platform for positively charged proteins or other analytes. Thus, the PDA functionalization of ZnO enables two approaches of its application in biosensors: (i) it could be used as a negatively charged layer which binds positively charged analytes (electrostatic interaction); and (ii) due to the presence of reactive groups on its surface, PDA film offers further biomolecule immobilization for biosensors and biochip construction (Wan-der-Waals interaction).

The formation of the ZnO–PDA interface was previously discussed in Fedorenko et al. [6]. Due to the proposed model, PDA formed on ZnO surface, and led to a reduced concentration of defects and enhanced the depletion layer width [6]. This resulted in the decrease in ZnO photoluminescence intensity and peak shift [6]. In the present paper, we observed similar behavior with an increase in PDA concentration. A significant change in PL intensity is already observed under a 0.3 mg/mL PDA concentration. Increasing the PDA concentrations leads to the decrease in the PL intensity that may be explained by the improvement of the PDA layer conformality, as was demonstrated by TEM analysis. FTIR and Raman showed the formation of additional hydroxyl groups on the ZnO/PDA interface. No significant influence of PDA concentration on the Raman and FTIR spectra was recorded. However, the enhancement of the PDA concentration might reduce the potential response of ZnO–PDA biosensor. 

In order to evaluate the correlation between the surface and optical properties of ZnO/PDA nanostructures, probe testing towards 50 μg/mL of poly-l-lysine (PLL) was performed. It is known, that poly-l-lysine contains positively charged hydrophilic amino groups even in moderately alkaline media [27,28]. It has been used for sensing [29,30], biomimetic [31], tissue engineering [32,33] and drug delivery applications [34,35].

**Figure 5:**
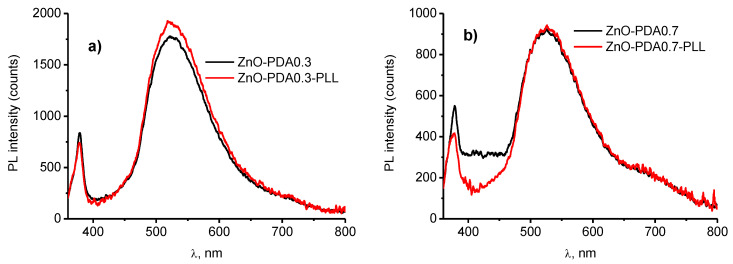

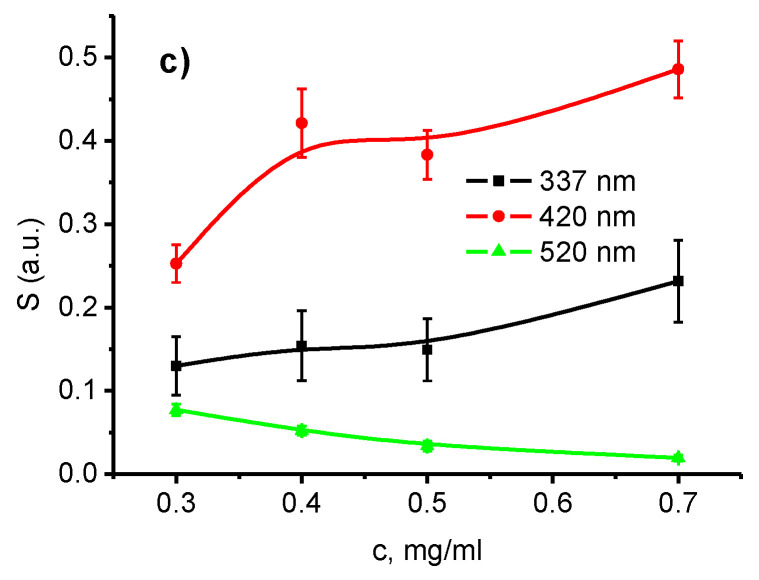
ZnO–PDA nanorods response to poly-l-lysine (PLL): (**a**) PL spectra of ZnO–PDA 0.3 mg/mL; (**b**) PL spectra of ZnO–PDA 0.7 mg/mL; and (**c**) response to PLL of ZnO–PDA with different PDA concentrations.

It was found that the PLL adsorption on the surface of ZnO/PDA resulted in the change in PL intensity (Figure 5a,b). The analysis of the change in PL intensity after the PLL adsorption was performed for fixed wavelengths of 377, 420 and 520 nm. The signal change was calculated as [3]

S = [1 − I(PLL)/I(0)],(1)

where I(0) and I(PLL) are the PL intensity before and after PLL deposition, respectively. It is seen from Figure 5a,b that PLL resulted in a PL intensity decrease in the range 370–430 nm and a PL increase in the range of 490–560 nm. PDA concentration was a key factor for response to PLL. The plotted signal change for the selected wavelengths was plotted in Figure 5c. It was found that the sensor response was the highest for 420 nm and it was significantly small for 520 nm. The signal response to PLL increased with the increase in PDA concentration.

It was assumed that positively charged PLL will passivate the negatively charged surface of ZnO/PDA. The enhancement of PL was expected due to the model proposed by Fedorenko et al. [6]. However, adsorbed PLL molecules on the surface of ZnO/PDA resulted in a decrease in UV peaks. We suppose that two competing processes might take place. As it is supposed that a non-conformal layer was obtained at low concentrations, the interaction between ZnO and PLL might take place. Previously, we showed that the deposition of a positively charged polymer over ZnO resulted in an increase in PL [22]. Thus, the interaction between PLL and PDA in the conformal coated areas could result in a decrease in UV emission and the interaction of PLL with ZnO in uncoated regions had an opposite effect. As with the increase in the PDA concentration, the coating quality increased, therefore the PL intensity in the visible range remains unchanged. 

## 4. Conclusions

In summary, we represented the first data on PDA concentration influence on ZnO–PDA nanocomposite properties and sensing. The correlation between the structural and optical properties of 1D ZnO–PDA nanostructures was evaluated. The detailed study of structural and optical properties of ZnO–PDA nanocomposites was represented. The TEM images demonstrated the ability to produce conformal PDA coating over ZnO nanorods after optimizing the concentration of precursors. Additionally, the progressive coverage of the PDA layer shows the gradual decrement in the optical bandgap of ZnO. The analysis of FTIR and Raman spectra after the formation of the ZnO–PDA composite, was shown. Based on these measurements (FTIR and Raman spectroscopy), it is suggested that PDA was attached to the ZnO via –OH groups. The interaction between ZnO–PDA and the model poly-l-lysine molecules showed the change of photoluminescence spectra in the UV and visible ranges. Changes in the emission intensity in the UV range are related to PLL–PDA interaction, whereas changes in visible spectra correspond to ZnO–PLL interaction. The ZnO–PLL interaction rate was suppressed at higher PDA concentrations (0.5 and 0.7 mg/mL).
